# (2*S*,3*R*,4*S*,4a*R*)-2,3,4,7-Tetra­hydroxy-3,4,4a,5-tetra­hydro­[1,3]dioxolo[4,5-*j*]phenanthridin-6(2*H*)-one hemihydrate

**DOI:** 10.1107/S1600536812048763

**Published:** 2012-12-05

**Authors:** Evgheni Jucov, Alexander Kornienko, Marco Masi, Antonio Evidente, Mikhail Antipin

**Affiliations:** a Institute of Applied Physics of the Academy of Sciences of Moldova, 5 Academy Street, MD-2028, Chisinau, Republic of Moldova; b Department of Chemistry and Biochemistry, Texas State University, 601 University Drive, San Marcos, TX 78666, USA; c Dipartimento di Scienze Chimiche, Università di Napoli Federico II, Complesso Universitario Monte S. Angelo, Via Cinthia 4, Napoli 80126, Italy; d Department of Chemistry & Biology, New Mexico Highlands University, 803 University Avenue, Las Vegas, NM 87701, USA

## Abstract

The title natural compound, isolated from *Narcissus pseudonarcissus* var. King Alfred crystallizes as a hemihydrate, C_14_H_13_NO_7_·0.5H_2_O, with four crystallographically independent dioxolophenanthridinone mol­ecules and two crystallographically independent solvent water mol­ecules in the asymmetric unit. All four crystallographically independent dioxolophenanthridinone mol­ecules are geometrically very similar and differ only in the orientations of the three hy­droxy groups at the terminal cyclo­hexene rings. The five-membered dioxolane ring has a planar conformation (the r.m.s. deviations are 0.010, 0.019, 0.025 and 0.004 Å, for the four crystallographically independent molecules), and the six-membered dihydro­pyridone and cyclo­hexene rings adopt sofa conformations in each mol­ecule. The flattened structure of each dioxolophenanthridinone mol­ecule is supported by a strong intra­molecular O—H⋯O hydrogen bond. The N atom has a slightly pyramidalized configuration. In the crystal, the dioxolophenanthridinone mol­ecules form layers parallel to (101) with O—H⋯O and N—H⋯O hydrogen bonds linking the dioxolophenanthridinone mol­ecules both within and between the layers and the water mol­ecules, forming a three-dimensional framework. The absolute configurations of the chiral centers are 2*S*, 3*R*, 4*S* and 4a*R*.

## Related literature

For general background to narciclasine, see: Ceriotti (1967*a* ▶,*b* ▶); Ceriotti *et al.* (1967 ▶); Kornienko & Evidente (2008 ▶). For the crystal structures of related compounds, see: Savona *et al.* (1970 ▶); Immirzi & Fuganti (1972 ▶); Bi *et al.* (1998 ▶); McNulty *et al.* (2011 ▶).
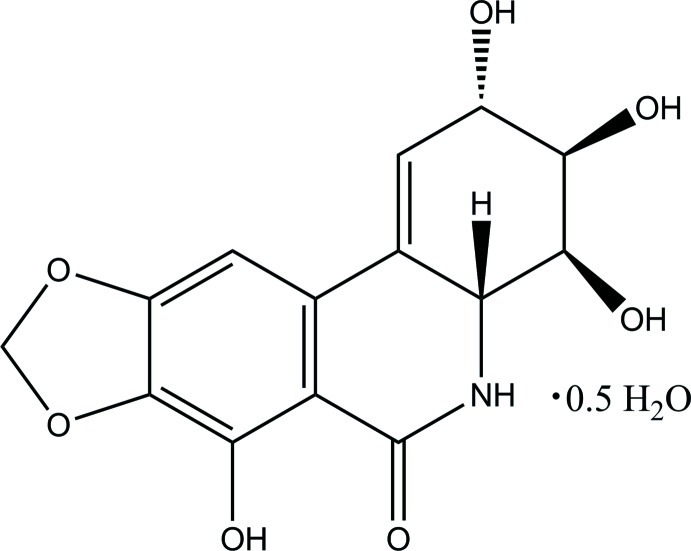


## Experimental

### 

#### Crystal data

C_14_H_13_NO_7_·0.5H_2_O
*M* *_r_* = 316.26
Monoclinic, 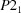

*a* = 10.90063 (8) Å
*b* = 20.37357 (17) Å
*c* = 11.88385 (9) Å
β = 104.3549 (8)°
*V* = 2556.82 (4) Å^3^
*Z* = 8
Cu *K*α radiation
μ = 1.16 mm^−1^
*T* = 100 K
0.25 × 0.23 × 0.06 mm

#### Data collection

Agilent SuperNova (Dual, Cu at zero, Atlas CCD) diffractometer
Absorption correction: multi-scan (*CrysAlis PRO*; Agilent, 2012 ▶) *T*_min_ = 0.738, *T*_max_ = 1.000
57161 measured reflections
10051 independent reflections
9997 reflections with *I* > 2σ(*I*)
*R*_int_ = 0.028

#### Refinement

*R*[*F* ^2^ > 2σ(*F* ^2^)] = 0.047
*wR*(*F* ^2^) = 0.122
*S* = 1.05
10051 reflections
908 parameters
1 restraint
H atoms treated by a mixture of independent and constrained refinement
Δρ_max_ = 0.27 e Å^−3^
Δρ_min_ = −0.21 e Å^−3^
Absolute structure: Flack (1983 ▶), 4707 Friedel pairs
Flack parameter: 0.05 (8)

### 

Data collection: *CrysAlis PRO* (Agilent, 2012 ▶); cell refinement: *CrysAlis PRO*; data reduction: *CrysAlis PRO*; program(s) used to solve structure: *SHELXTL* (Sheldrick, 2008 ▶); program(s) used to refine structure: *SHELXTL*; molecular graphics: *SHELXTL*; software used to prepare material for publication: *SHELXTL*.

## Supplementary Material

Crystal structure: contains datablock(s) global, I. DOI: 10.1107/S1600536812048763/rk2387sup1.cif

Structure factors: contains datablock(s) I. DOI: 10.1107/S1600536812048763/rk2387Isup2.hkl

Supplementary material file. DOI: 10.1107/S1600536812048763/rk2387Isup3.cml

Additional supplementary materials:  crystallographic information; 3D view; checkCIF report

## Figures and Tables

**Table 1:** Hydrogen-bond geometry (Å, °)

*D*—H⋯*A*	*D*—H	H⋯*A*	*D*⋯*A*	*D*—H⋯*A*
O3—H3⋯O4	0.96 (3)	1.57 (4)	2.4842 (19)	158 (3)
O5—H5⋯O7*A* ^i^	0.84 (4)	1.88 (4)	2.698 (2)	166 (4)
O6—H6⋯O3*A* ^ii^	0.85 (3)	1.98 (3)	2.745 (2)	150 (3)
O7—H7⋯O7*C* ^iii^	0.89 (3)	1.89 (3)	2.768 (2)	170 (3)
N1—H1⋯O4*B* ^i^	0.91 (4)	2.25 (4)	3.105 (2)	157 (3)
O3*A*—H3*A*⋯O4*A*	1.00 (4)	1.55 (4)	2.4656 (19)	151 (4)
O5*A*—H5*A*⋯O7	0.87 (3)	2.23 (3)	3.050 (2)	157 (3)
O6*A*—H6*AA*⋯O1^ii^	0.82 (4)	2.15 (4)	2.906 (2)	153 (3)
O7*A*—H7*A*⋯O1*A* ^iv^	0.83 (4)	2.29 (4)	2.901 (2)	131 (3)
N1*A*—H1*AA*⋯O4*C*	0.89 (3)	1.91 (3)	2.796 (2)	171 (3)
O3*B*—H3*B*⋯O4*B*	1.01 (4)	1.57 (4)	2.537 (2)	159 (4)
O5*B*—H5*B*⋯O2*W* ^v^	0.94 (4)	1.80 (4)	2.701 (2)	159 (4)
O6*B*—H6*B*⋯O6^vi^	0.89 (4)	2.03 (4)	2.8857 (19)	163 (3)
O7*B*—H7*B*⋯O5^vii^	0.86 (4)	1.86 (4)	2.714 (2)	175 (4)
N1*B*—H1*BA*⋯O4^v^	0.89 (4)	1.99 (4)	2.870 (2)	171 (3)
O3*C*—H3*C*⋯O4*C*	0.95 (5)	1.59 (5)	2.4797 (18)	154 (5)
O5*C*—H5*C*⋯O6*B*	0.82 (3)	2.01 (4)	2.829 (2)	175 (3)
O6*C*—H6*C*⋯O6*A* ^vi^	0.88 (4)	1.93 (3)	2.7893 (19)	165 (3)
O7*C*—H7*C*⋯O3*C* ^vii^	0.86 (4)	1.93 (4)	2.767 (2)	162 (3)
N1*C*—H1*C*⋯O4*A*	0.91 (4)	2.03 (4)	2.911 (2)	164 (3)
O1*W*—H1*WA*⋯O7*B* ^i^	0.90 (4)	1.94 (4)	2.835 (2)	173 (4)
O1*W*—H1*WB*⋯O5*A* ^vii^	0.89 (5)	2.22 (5)	3.036 (2)	153 (4)
O2*W*—H2*WA*⋯O1*W*	0.90 (3)	1.94 (3)	2.833 (2)	167 (3)
O2*W*—H2WB⋯O6*C*	0.87 (4)	2.00 (4)	2.869 (2)	172 (3)

Symmetry codes: (i) 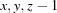; (ii) 
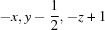
; (iii) 
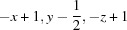
; (iv) 
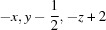
; (v) 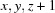; (vi) 
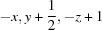
; (vii) 
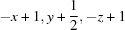
.

**Fig. 1.**
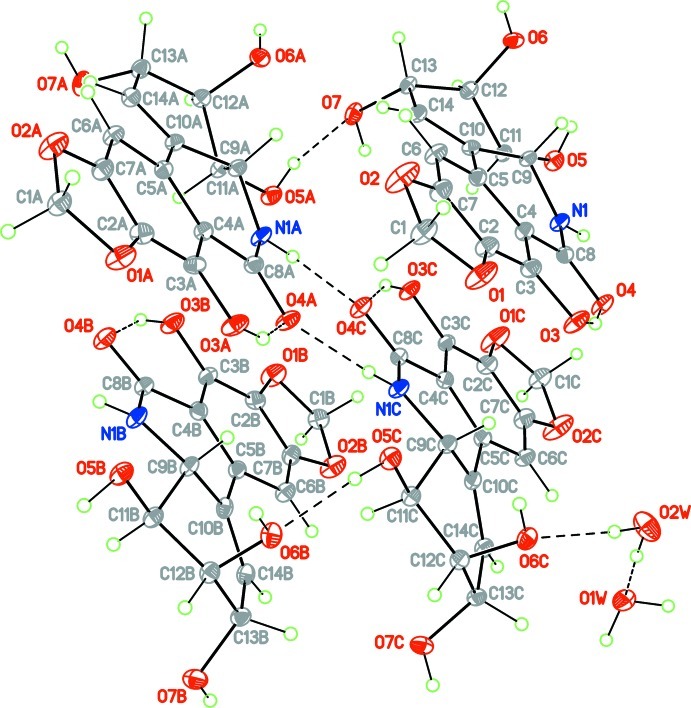
Molecular structure with the atom numbering scheme of I (four crystallographically independent molecules and two water solvate molecules are presented). Displacement ellipsoids are shown at the 50% probability level. H atoms are presented as small spheres of arbitrary radius. Dashed lines indicate the O–H···O and N–H···O hydrogen bonds.

**Fig. 2.**
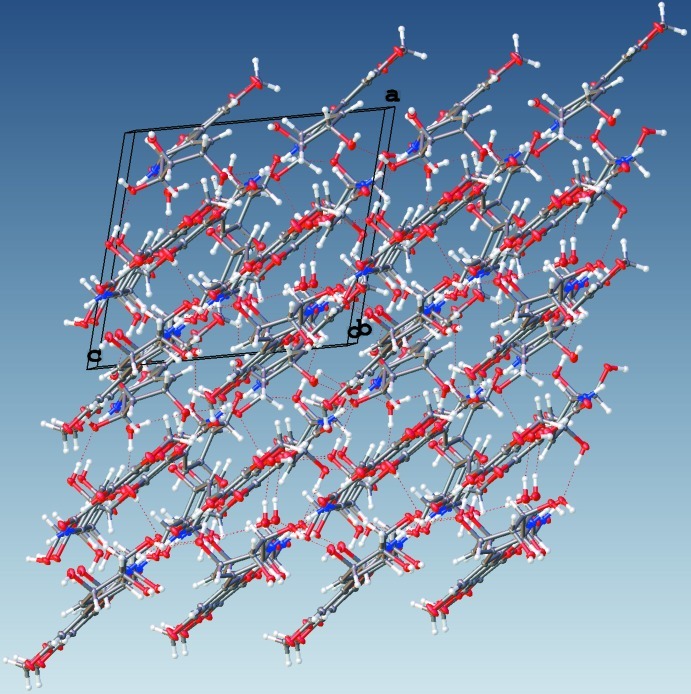
Crystal packing of I. Dashed lines indicate the O–H···O and N–H···O hydrogen bonds.

## supplementary crystallographic information

### Comment

Since his first isolation from *Narcissus* bulbs by Ceriotti in 1967 (Ceriotti, 1967*a*; Ceriotti, 1967*b*; Ceriotti *et al.*, 1967), narciclasine has been extensively studied from chemical and biological standpoints (Kornienko & Evidente, 2008). It exhibits a broad range of biological activities and thus has various potential practical applications, for example, in the treatment of brain cancers, such as glioblastoma multiforme. The structure and absolute stereochemistry of this isocarbostyril related to the *Amaryllidaceae* alkaloids was unambiguously elucidated by an X-ray analysis of the corresponding tetraacetate (Savona *et al.*, 1970; Immirzi & Fuganti, 1972). In 1998, narciclasine isolated from the mucilage of *Narcissustazetta L*. bulbs was studied by an X-ray diffraction analysis, but the crystallographic data and structure refinement details were not presented and deposited (Bi *et al.*, 1998). Furthermore, the X-ray data of *cis*-dihydronarciclasine were reported very recently (McNulty *et al.*, 2011). Because of its biological importance, narciclasine is studied in complexes with the target proteins. To assist in these investigations we prepared crystalline natural product and performed an X-ray single-crystal analysis.

The title compound, I, crystallizes as hemihydrate, *i.e.*, C_14_H_13_NO_7_^.^0.5H_2_O, with the four crystallographically independent molecules of I and the two crystallographically independent water solvate molecules in the unit cell (Fig. 1). All the four crystallographically independent molecules of I are geometrically very similar and differ only by the orientations of the three hydroxy groups at the terminal cyclohexene rings.

The molecule of I comprises a fused tetracyclic system containing one five-membered ring (dioxolane) and three six-membered rings (benzene, dihydropyridone and cyclohexene) (Fig. 1). The five-membered dioxolane ring has the planar conformation, and the six-membered dihydropyridone and cyclohexene rings adopt the *sofa* conformations. In the case of dihydropyridone ring, the bridged carbon atom adjacent to the nitrogen atom deviates from the plane passed through the other atoms of the ring, and, in the case of cyclohexene ring, the carbon atom bearing the hydroxy group and adjacent to the bridged carbon atom is out of the plane passed through the other atoms of the ring. The flattened structure of molecule of I is supported by the strong intramolecular O–H···O hydrogen bond (Table 1). The nitrogen atom has a slightly pyramidalized configuration.

The different disposition of the hydrogen atoms of the hydroxy groups in the four crystallographically independent molecules of I as well as the observed configurations of the nitrogen atoms is explained by developed hydrogen bonding system in the crystal of I (Table 1). In the crystal, the molecules of I form the layers parallel to (101) (Fig. 2). The intermolecular O–H···O and N–H···O hydrogen bonds link the molecules of I both within and between the layers and water solvate molecules into three-dimensional framework (Fig. 2).

The molecule of I possesses four asymmetric centers at the quaternary carbon atoms of the terminal cyclohexene ring. The absolute structure of I was objectively determined by use of Cu *K*α radiation and the refinement of Flack parameter. The absolute configurations of the chiral centers are 2*S*,3*R*,4*S*,4a*R*.

### Experimental

Narciclasine, I, was isolated from the dried and minced bulbs of *Narcissus pseudonarcissus* var. *King Alfred* using an alternative method compared to those reported previously (Kornienko & Evidente, 2008). The methanol organic extract obtained using a Soxhlet was purified with two steps of column flash chromatography and 1 g of I was collected as amorphous solid. White single crystals of I suitable for an X-ray analysis were obtained by a slow dissolution of water in narciclasine-containing *DMSO*.

### Refinement

The hydrogen atoms of the hydroxy- and amino-groups were localized in the difference Fourier map and refined isotropically. The other hydrogen atoms were placed in calculated positions with C–H = 0.95-1.00Å and refined in the riding model with fixed isotropic displacement parameters with *U*_iso_(H) = 1.2*U*_eq_(C).

The absolute structure of I was objectively determined by the refinement of Flack parameter, which has become equal to 0.05 (8).

### Crystal data

**Table:**

C_14_H_13_NO_7_·0.5H_2_O	*F*(000) = 1320
*M**_r_* = 316.26	*D*_x_ = 1.643 Mg m^−^^3^
Monoclinic, *P*2_1_	Cu *K*α radiation, λ = 1.5418 Å
Hall symbol: P 2yb	Cell parameters from 36580 reflections
*a* = 10.90063 (8) Å	θ = 3.8–73.8°
*b* = 20.37357 (17) Å	µ = 1.16 mm^−^^1^
*c* = 11.88385 (9) Å	*T* = 100 K
β = 104.3549 (8)°	Plate, colourless
*V* = 2556.82 (4) Å^3^	0.25 × 0.23 × 0.06 mm
*Z* = 8	

### Data collection

**Table:**

Agilent SuperNova (Dual, Cu at zero, Atlas CCD) diffractometer	10051 independent reflections
Radiation source: SuperNova (Cu) X-ray Source	9997 reflections with *I* > 2σ(*I*)
Mirror monochromator	*R*_int_ = 0.028
Detector resolution: 10.3756 pixels mm^-1^	θ_max_ = 74.0°, θ_min_ = 3.8°
ω–scans	*h* = −13→13
Absorption correction: multi-scan (*CrysAlis PRO*; Agilent, 2012)	*k* = −25→23
*T*_min_ = 0.738, *T*_max_ = 1.000	*l* = −14→14
57161 measured reflections	

### Refinement

**Table:**

Refinement on *F*^2^	Hydrogen site location: difference Fourier map
Least-squares matrix: full	H atoms treated by a mixture of independent and constrained refinement
*R*[*F*^2^ > 2σ(*F*^2^)] = 0.047	*w* = 1/[σ^2^(*F*_o_^2^) + (0.1041*P*)^2^ + 0.4322*P*] where *P* = (*F*_o_^2^ + 2*F*_c_^2^)/3
*wR*(*F*^2^) = 0.122	(Δ/σ)_max_ < 0.001
*S* = 1.05	Δρ_max_ = 0.27 e Å^−^^3^
10051 reflections	Δρ_min_ = −0.21 e Å^−^^3^
908 parameters	Extinction correction: *SHELXTL* (Sheldrick, 2008), Fc^*^=kFc[1+0.001xFc^2^λ^3^/sin(2θ)]^-1/4^
1 restraint	Extinction coefficient: 0.00102 (16)
Primary atom site location: structure-invariant direct methods	Absolute structure: Flack (1983), 4707 Friedel pairs
Secondary atom site location: difference Fourier map	Flack parameter: 0.05 (8)

### Special details

**Table:**

Geometry. All s.u.'s (except the s.u. in the dihedral angle between two l.s. planes) are estimated using the full covariance matrix. The cell s.u.'s are taken into account individually in the estimation of s.u.'s in distances, angles and torsion angles; correlations between s.u.'s in cell parameters are only used when they are defined by crystal symmetry. An approximate (isotropic) treatment of cell s.u.'s is used for estimating s.u.'s involving l.s. planes.
Refinement. Refinement of *F*^2^ against ALL reflections. The weighted *R*-factor *wR* and goodness of fit *S* are based on *F*^2^, conventional *R*-factors *R* are based on *F*, with *F* set to zero for negative *F*^2^. The threshold expression of *F*^2^ > σ(*F*^2^) is used only for calculating *R*-factors(gt) *etc*. and is not relevant to the choice of reflections for refinement. *R*-factors based on *F*^2^ are statistically about twice as large as those based on *F*, and *R*-factors based on ALL data will be even larger.

### Fractional atomic coordinates and isotropic or equivalent isotropic displacement parameters (Å^2^)

**Table:**

	*x*	*y*	*z*	*U*_iso_*/*U*_eq_	
O1	−0.10128 (16)	0.58845 (7)	0.40287 (15)	0.0248 (3)	
O2	−0.16470 (17)	0.49383 (8)	0.48170 (15)	0.0280 (4)	
O3	0.05191 (14)	0.58470 (7)	0.23220 (13)	0.0186 (3)	
H3	0.103 (3)	0.5663 (17)	0.185 (3)	0.033 (8)*	
O4	0.17583 (14)	0.51247 (7)	0.13295 (12)	0.0173 (3)	
O5	0.24849 (13)	0.27851 (7)	0.07456 (12)	0.0153 (3)	
H5	0.203 (4)	0.253 (2)	0.027 (4)	0.056 (11)*	
O6	0.03231 (13)	0.21287 (7)	0.12059 (12)	0.0161 (3)	
H6	0.013 (3)	0.1724 (16)	0.118 (2)	0.022 (7)*	
O7	0.22117 (13)	0.21004 (7)	0.42339 (12)	0.0173 (3)	
H7	0.292 (3)	0.2276 (16)	0.413 (3)	0.032 (8)*	
N1	0.17149 (15)	0.40217 (8)	0.14336 (14)	0.0133 (3)	
H1	0.222 (3)	0.3984 (16)	0.094 (3)	0.032 (8)*	
C1	−0.1697 (2)	0.56383 (10)	0.48424 (18)	0.0187 (4)	
H1A	−0.1304	0.5801	0.5635	0.022*	
H1B	−0.2588	0.5789	0.4618	0.022*	
C2	−0.05465 (18)	0.53344 (10)	0.36023 (17)	0.0150 (4)	
C3	0.01784 (18)	0.52988 (10)	0.28006 (17)	0.0143 (4)	
C4	0.05381 (17)	0.46632 (10)	0.25195 (16)	0.0123 (4)	
C5	0.01637 (17)	0.40965 (10)	0.30334 (15)	0.0124 (4)	
C6	−0.06126 (18)	0.41495 (10)	0.38108 (17)	0.0150 (4)	
H6A	−0.0907	0.3773	0.4137	0.018*	
C7	−0.09250 (18)	0.47744 (11)	0.40765 (17)	0.0167 (4)	
C8	0.13693 (17)	0.46133 (9)	0.17180 (15)	0.0123 (4)	
C9	0.11265 (16)	0.34097 (9)	0.16808 (15)	0.0109 (3)	
H9	0.0386	0.3315	0.1014	0.013*	
C10	0.06533 (16)	0.34561 (10)	0.27760 (15)	0.0113 (3)	
C11	0.20704 (16)	0.28453 (9)	0.17845 (15)	0.0115 (3)	
H11	0.2824	0.2949	0.2434	0.014*	
C12	0.14720 (17)	0.22178 (9)	0.20911 (15)	0.0128 (3)	
H12	0.2054	0.1838	0.2089	0.015*	
C13	0.11638 (17)	0.22692 (10)	0.32783 (15)	0.0129 (3)	
H13	0.0470	0.1948	0.3280	0.015*	
C14	0.06725 (16)	0.29371 (10)	0.34676 (15)	0.0128 (3)	
H14	0.0349	0.2996	0.4133	0.015*	
O1A	−0.17618 (14)	0.57428 (7)	0.96267 (13)	0.0192 (3)	
O2A	−0.24300 (15)	0.47333 (8)	1.01724 (14)	0.0212 (3)	
O3A	−0.01378 (14)	0.58673 (7)	0.80411 (13)	0.0173 (3)	
H3A	0.049 (3)	0.5769 (19)	0.758 (3)	0.048 (10)*	
O4A	0.11829 (13)	0.52761 (7)	0.69631 (12)	0.0161 (3)	
O5A	0.24668 (12)	0.30645 (7)	0.62426 (12)	0.0153 (3)	
H5A	0.217 (3)	0.2783 (17)	0.569 (3)	0.030 (8)*	
O6A	0.02956 (12)	0.22587 (8)	0.59640 (11)	0.0151 (3)	
H6AA	0.028 (3)	0.1870 (18)	0.578 (3)	0.037 (9)*	
O7A	0.13673 (14)	0.19497 (8)	0.90368 (12)	0.0185 (3)	
H7A	0.100 (4)	0.163 (2)	0.923 (3)	0.051 (10)*	
N1A	0.12678 (15)	0.41726 (8)	0.68905 (14)	0.0134 (3)	
H1AA	0.175 (3)	0.4229 (17)	0.640 (3)	0.031 (8)*	
C1A	−0.24805 (18)	0.54300 (10)	1.03442 (17)	0.0157 (4)	
H1AB	−0.2116	0.5540	1.1171	0.019*	
H1AC	−0.3370	0.5584	1.0124	0.019*	
C2A	−0.12403 (18)	0.52349 (10)	0.91416 (17)	0.0143 (4)	
C3A	−0.04642 (17)	0.52784 (10)	0.83915 (16)	0.0128 (4)	
C4A	−0.00376 (17)	0.46811 (10)	0.80138 (15)	0.0117 (4)	
C5A	−0.04178 (17)	0.40713 (10)	0.83707 (15)	0.0115 (4)	
C6A	−0.12623 (18)	0.40417 (10)	0.90945 (16)	0.0134 (4)	
H6AB	−0.1564	0.3636	0.9313	0.016*	
C7A	−0.16278 (17)	0.46344 (10)	0.94683 (16)	0.0145 (4)	
C8A	0.08361 (17)	0.47205 (10)	0.72538 (16)	0.0124 (4)	
C9A	0.07358 (17)	0.35238 (10)	0.69731 (15)	0.0120 (3)	
H9A	0.0084	0.3436	0.6233	0.014*	
C10A	0.01006 (16)	0.34682 (10)	0.79716 (15)	0.0118 (3)	
C11A	0.17709 (16)	0.30016 (10)	0.71063 (15)	0.0125 (3)	
H11A	0.2374	0.3062	0.7884	0.015*	
C12A	0.11766 (17)	0.23275 (9)	0.70834 (15)	0.0125 (4)	
H12A	0.1851	0.1985	0.7169	0.015*	
C13A	0.04951 (17)	0.22481 (10)	0.80618 (16)	0.0140 (4)	
H13A	−0.0242	0.1946	0.7788	0.017*	
C14A	0.00246 (17)	0.28815 (10)	0.84499 (15)	0.0128 (4)	
H14A	−0.0357	0.2863	0.9086	0.015*	
O1B	0.60207 (15)	0.38711 (8)	0.67445 (14)	0.0224 (3)	
O2B	0.66136 (15)	0.48840 (8)	0.61543 (13)	0.0215 (3)	
O3B	0.45297 (15)	0.37256 (7)	0.84392 (13)	0.0207 (3)	
H3B	0.405 (4)	0.386 (2)	0.902 (4)	0.055 (11)*	
O4B	0.34387 (14)	0.43363 (7)	0.97790 (13)	0.0185 (3)	
O5B	0.19712 (12)	0.65451 (7)	1.02628 (12)	0.0160 (3)	
H5B	0.212 (4)	0.679 (2)	1.095 (4)	0.058 (11)*	
O6B	0.20914 (13)	0.73271 (7)	0.82583 (11)	0.0161 (3)	
H6B	0.137 (3)	0.7348 (18)	0.847 (3)	0.043 (9)*	
O7B	0.52571 (13)	0.76627 (7)	0.98119 (12)	0.0184 (3)	
H7B	0.596 (4)	0.768 (2)	0.962 (3)	0.050 (10)*	
N1B	0.31833 (16)	0.54343 (9)	0.96672 (14)	0.0155 (3)	
H1BA	0.277 (3)	0.5380 (17)	1.022 (3)	0.037 (8)*	
C1B	0.67792 (18)	0.41867 (10)	0.60769 (17)	0.0171 (4)	
H1BB	0.6512	0.4043	0.5256	0.021*	
H1BC	0.7682	0.4069	0.6385	0.021*	
C2B	0.55542 (18)	0.43707 (10)	0.72953 (17)	0.0143 (4)	
C3B	0.48186 (18)	0.43187 (10)	0.80840 (16)	0.0150 (4)	
C4B	0.44111 (17)	0.49147 (10)	0.84873 (15)	0.0131 (4)	
C5B	0.47705 (17)	0.55285 (10)	0.81149 (15)	0.0125 (4)	
C6B	0.55311 (18)	0.55598 (10)	0.73210 (17)	0.0144 (4)	
H6BA	0.5780	0.5968	0.7059	0.017*	
C7B	0.58981 (18)	0.49713 (11)	0.69404 (16)	0.0151 (4)	
C8B	0.36408 (17)	0.48781 (10)	0.93492 (16)	0.0142 (4)	
C9B	0.31785 (17)	0.60607 (10)	0.90653 (16)	0.0126 (4)	
H9B	0.2407	0.6077	0.8403	0.015*	
C10B	0.43332 (17)	0.61301 (10)	0.85803 (15)	0.0125 (4)	
C11B	0.31061 (17)	0.66210 (10)	0.98879 (15)	0.0131 (4)	
H11B	0.3849	0.6597	1.0577	0.016*	
C12B	0.31304 (17)	0.72767 (10)	0.92712 (16)	0.0134 (4)	
H12B	0.3067	0.7641	0.9818	0.016*	
C13B	0.43532 (17)	0.73580 (9)	0.88773 (15)	0.0134 (4)	
H13B	0.4183	0.7662	0.8195	0.016*	
C14B	0.48536 (17)	0.67210 (10)	0.85247 (15)	0.0134 (4)	
H14B	0.5595	0.6740	0.8241	0.016*	
O1C	0.53886 (17)	0.38983 (8)	0.24948 (15)	0.0276 (4)	
O2C	0.60370 (18)	0.48792 (8)	0.18242 (15)	0.0289 (4)	
O3C	0.38164 (13)	0.38340 (7)	0.41199 (12)	0.0158 (3)	
H3C	0.332 (5)	0.396 (2)	0.464 (4)	0.074 (14)*	
O4C	0.27433 (14)	0.44915 (7)	0.53502 (12)	0.0167 (3)	
O5C	0.17104 (12)	0.67618 (7)	0.60239 (12)	0.0147 (3)	
H5C	0.182 (3)	0.6905 (17)	0.669 (3)	0.034 (8)*	
O6C	0.22150 (12)	0.76137 (7)	0.42056 (11)	0.0149 (3)	
H6C	0.146 (3)	0.7503 (17)	0.429 (3)	0.038 (8)*	
O7C	0.54366 (12)	0.75389 (7)	0.59326 (12)	0.0159 (3)	
H7C	0.553 (3)	0.7959 (18)	0.598 (2)	0.026 (7)*	
N1C	0.26085 (15)	0.55955 (8)	0.52635 (14)	0.0136 (3)	
H1C	0.211 (3)	0.5576 (18)	0.577 (3)	0.039 (9)*	
C1C	0.6107 (2)	0.41765 (11)	0.17460 (18)	0.0188 (4)	
H1CA	0.7000	0.4030	0.1992	0.023*	
H1CB	0.5753	0.4032	0.0935	0.023*	
C2C	0.49214 (19)	0.44261 (10)	0.29700 (16)	0.0149 (4)	
C3C	0.41672 (17)	0.44140 (10)	0.37436 (16)	0.0127 (4)	
C4C	0.38239 (17)	0.50309 (10)	0.41249 (15)	0.0116 (4)	
C5C	0.42654 (17)	0.56230 (10)	0.37607 (16)	0.0128 (4)	
C6C	0.50168 (18)	0.56195 (10)	0.29527 (16)	0.0147 (4)	
H6CA	0.5302	0.6014	0.2675	0.018*	
C7C	0.53138 (19)	0.50111 (11)	0.25880 (17)	0.0169 (4)	
C8C	0.30235 (17)	0.50300 (10)	0.49545 (15)	0.0126 (4)	
C9C	0.27952 (17)	0.62357 (9)	0.47638 (15)	0.0122 (3)	
H9C	0.2044	0.6331	0.4107	0.015*	
C10C	0.39702 (17)	0.62355 (10)	0.43001 (16)	0.0121 (3)	
C11C	0.28799 (16)	0.67616 (10)	0.56871 (15)	0.0122 (3)	
H11C	0.3581	0.6647	0.6377	0.015*	
C12C	0.31542 (17)	0.74292 (9)	0.52269 (15)	0.0128 (3)	
H12C	0.3185	0.7768	0.5843	0.015*	
C13C	0.44355 (16)	0.74143 (9)	0.49068 (15)	0.0127 (3)	
H13C	0.4444	0.7770	0.4328	0.015*	
C14C	0.47034 (16)	0.67711 (9)	0.43991 (15)	0.0131 (3)	
H14C	0.5451	0.6741	0.4127	0.016*	
O1W	0.53702 (14)	0.73287 (8)	0.21514 (14)	0.0222 (3)	
H1WA	0.533 (3)	0.7470 (19)	0.142 (3)	0.046 (9)*	
H1WB	0.578 (4)	0.762 (2)	0.265 (4)	0.070 (13)*	
O2W	0.27386 (15)	0.74364 (9)	0.19752 (13)	0.0257 (3)	
H2WA	0.359 (3)	0.7453 (15)	0.212 (2)	0.024 (7)*	

### Atomic displacement parameters (Å^2^)

**Table:**

	*U*^11^	*U*^22^	*U*^33^	*U*^12^	*U*^13^	*U*^23^
O1	0.0383 (9)	0.0097 (8)	0.0359 (8)	0.0010 (6)	0.0274 (7)	−0.0011 (6)
O2	0.0433 (9)	0.0121 (8)	0.0414 (9)	0.0030 (7)	0.0345 (8)	−0.0008 (7)
O3	0.0263 (7)	0.0084 (7)	0.0260 (7)	0.0003 (5)	0.0156 (6)	0.0021 (5)
O4	0.0246 (7)	0.0108 (7)	0.0206 (7)	−0.0009 (5)	0.0137 (5)	0.0024 (5)
O5	0.0172 (6)	0.0158 (7)	0.0158 (6)	0.0015 (5)	0.0096 (5)	−0.0016 (5)
O6	0.0183 (7)	0.0112 (7)	0.0184 (6)	−0.0048 (5)	0.0041 (5)	−0.0021 (5)
O7	0.0161 (6)	0.0192 (8)	0.0173 (6)	0.0032 (5)	0.0054 (5)	0.0079 (5)
N1	0.0163 (7)	0.0103 (8)	0.0157 (7)	−0.0007 (6)	0.0084 (6)	0.0002 (6)
C1	0.0254 (10)	0.0116 (10)	0.0239 (9)	0.0021 (8)	0.0149 (8)	0.0002 (8)
C2	0.0186 (9)	0.0087 (10)	0.0194 (9)	0.0014 (7)	0.0082 (7)	−0.0018 (7)
C3	0.0167 (8)	0.0097 (9)	0.0166 (8)	−0.0003 (7)	0.0046 (7)	0.0000 (7)
C4	0.0140 (8)	0.0099 (9)	0.0135 (8)	0.0009 (7)	0.0047 (6)	−0.0002 (7)
C5	0.0134 (8)	0.0122 (10)	0.0119 (8)	−0.0004 (7)	0.0037 (6)	0.0000 (7)
C6	0.0179 (8)	0.0104 (9)	0.0198 (9)	0.0004 (7)	0.0105 (7)	0.0009 (7)
C7	0.0205 (9)	0.0154 (10)	0.0177 (9)	−0.0004 (7)	0.0114 (7)	−0.0001 (7)
C8	0.0146 (8)	0.0101 (9)	0.0118 (8)	−0.0006 (7)	0.0029 (6)	0.0000 (7)
C9	0.0134 (8)	0.0076 (9)	0.0129 (8)	−0.0009 (6)	0.0055 (6)	0.0005 (6)
C10	0.0106 (7)	0.0106 (9)	0.0136 (8)	−0.0014 (6)	0.0047 (6)	−0.0014 (7)
C11	0.0117 (8)	0.0118 (9)	0.0123 (8)	0.0005 (6)	0.0054 (6)	0.0007 (6)
C12	0.0135 (8)	0.0104 (9)	0.0155 (8)	0.0018 (7)	0.0059 (6)	0.0017 (7)
C13	0.0130 (8)	0.0123 (9)	0.0144 (8)	0.0000 (7)	0.0055 (6)	0.0033 (7)
C14	0.0137 (8)	0.0122 (9)	0.0137 (8)	−0.0007 (7)	0.0057 (6)	0.0002 (7)
O1A	0.0249 (7)	0.0109 (7)	0.0281 (7)	−0.0004 (6)	0.0182 (6)	−0.0047 (6)
O2A	0.0290 (7)	0.0135 (8)	0.0289 (8)	0.0005 (6)	0.0221 (6)	−0.0028 (6)
O3A	0.0228 (7)	0.0075 (7)	0.0257 (7)	−0.0006 (5)	0.0139 (6)	−0.0013 (5)
O4A	0.0215 (7)	0.0085 (7)	0.0220 (6)	−0.0015 (5)	0.0127 (5)	−0.0005 (5)
O5A	0.0166 (6)	0.0151 (7)	0.0177 (6)	−0.0017 (5)	0.0106 (5)	−0.0017 (5)
O6A	0.0177 (6)	0.0129 (7)	0.0150 (6)	−0.0016 (5)	0.0043 (5)	−0.0034 (5)
O7A	0.0279 (7)	0.0112 (7)	0.0169 (7)	0.0050 (6)	0.0066 (5)	0.0047 (5)
N1A	0.0182 (7)	0.0087 (8)	0.0171 (7)	−0.0006 (6)	0.0115 (6)	0.0002 (6)
C1A	0.0192 (9)	0.0135 (10)	0.0176 (8)	0.0011 (7)	0.0105 (7)	−0.0012 (7)
C2A	0.0153 (8)	0.0116 (10)	0.0173 (8)	0.0008 (7)	0.0063 (7)	−0.0036 (7)
C3A	0.0139 (8)	0.0084 (9)	0.0161 (8)	−0.0004 (7)	0.0036 (6)	−0.0011 (7)
C4A	0.0125 (8)	0.0109 (9)	0.0123 (8)	0.0000 (7)	0.0042 (6)	−0.0007 (7)
C5A	0.0118 (8)	0.0109 (10)	0.0119 (8)	−0.0002 (6)	0.0031 (6)	0.0004 (7)
C6A	0.0150 (8)	0.0106 (9)	0.0161 (8)	−0.0002 (7)	0.0068 (7)	0.0014 (7)
C7A	0.0140 (8)	0.0158 (10)	0.0155 (9)	0.0004 (7)	0.0071 (7)	0.0002 (7)
C8A	0.0134 (8)	0.0105 (9)	0.0136 (8)	−0.0010 (7)	0.0038 (6)	0.0000 (7)
C9A	0.0142 (8)	0.0086 (9)	0.0139 (8)	−0.0007 (7)	0.0050 (6)	0.0006 (7)
C10A	0.0111 (8)	0.0115 (9)	0.0133 (8)	−0.0014 (6)	0.0039 (6)	−0.0003 (7)
C11A	0.0149 (8)	0.0105 (9)	0.0135 (8)	0.0002 (7)	0.0064 (6)	0.0011 (7)
C12A	0.0145 (8)	0.0100 (9)	0.0140 (8)	0.0006 (7)	0.0052 (6)	−0.0012 (6)
C13A	0.0173 (8)	0.0102 (9)	0.0152 (8)	0.0000 (7)	0.0052 (7)	0.0009 (7)
C14A	0.0138 (8)	0.0120 (9)	0.0135 (8)	0.0006 (7)	0.0051 (6)	−0.0006 (7)
O1B	0.0318 (8)	0.0134 (7)	0.0282 (8)	0.0006 (6)	0.0189 (6)	−0.0040 (6)
O2B	0.0281 (7)	0.0150 (7)	0.0287 (7)	0.0007 (6)	0.0208 (6)	−0.0029 (6)
O3B	0.0303 (8)	0.0104 (7)	0.0251 (7)	−0.0025 (6)	0.0141 (6)	0.0002 (6)
O4B	0.0251 (7)	0.0129 (8)	0.0212 (7)	−0.0015 (6)	0.0130 (5)	0.0017 (5)
O5B	0.0155 (6)	0.0168 (7)	0.0193 (7)	−0.0002 (5)	0.0113 (5)	0.0010 (5)
O6B	0.0154 (6)	0.0186 (7)	0.0153 (6)	0.0031 (5)	0.0055 (5)	0.0023 (5)
O7B	0.0190 (7)	0.0203 (8)	0.0191 (7)	−0.0068 (5)	0.0108 (5)	−0.0068 (5)
N1B	0.0187 (8)	0.0137 (9)	0.0176 (7)	−0.0009 (6)	0.0112 (6)	0.0028 (6)
C1B	0.0177 (8)	0.0162 (10)	0.0194 (9)	0.0012 (7)	0.0082 (7)	−0.0028 (8)
C2B	0.0167 (8)	0.0112 (9)	0.0158 (8)	0.0012 (7)	0.0051 (7)	−0.0032 (7)
C3B	0.0178 (8)	0.0121 (10)	0.0157 (8)	−0.0024 (7)	0.0050 (7)	0.0013 (7)
C4B	0.0131 (8)	0.0145 (10)	0.0120 (8)	−0.0004 (7)	0.0039 (6)	−0.0005 (7)
C5B	0.0120 (8)	0.0132 (10)	0.0125 (8)	−0.0005 (7)	0.0032 (6)	0.0007 (7)
C6B	0.0160 (8)	0.0126 (10)	0.0165 (8)	0.0013 (7)	0.0078 (7)	0.0009 (7)
C7B	0.0152 (8)	0.0174 (10)	0.0145 (8)	0.0001 (7)	0.0070 (7)	0.0001 (7)
C8B	0.0144 (8)	0.0142 (10)	0.0147 (8)	−0.0023 (7)	0.0048 (6)	0.0014 (7)
C9B	0.0141 (8)	0.0113 (9)	0.0141 (8)	0.0002 (6)	0.0068 (6)	0.0022 (7)
C10B	0.0116 (8)	0.0159 (10)	0.0109 (8)	0.0019 (7)	0.0046 (6)	0.0022 (7)
C11B	0.0125 (8)	0.0145 (9)	0.0148 (8)	−0.0001 (7)	0.0080 (6)	0.0011 (7)
C12B	0.0145 (8)	0.0134 (9)	0.0135 (8)	0.0011 (7)	0.0057 (6)	0.0003 (7)
C13B	0.0155 (8)	0.0125 (9)	0.0142 (8)	−0.0021 (7)	0.0075 (6)	−0.0013 (7)
C14B	0.0139 (8)	0.0157 (10)	0.0121 (8)	0.0011 (7)	0.0061 (6)	0.0005 (7)
O1C	0.0464 (10)	0.0102 (8)	0.0385 (9)	0.0022 (7)	0.0336 (8)	−0.0022 (6)
O2C	0.0463 (9)	0.0135 (8)	0.0405 (9)	−0.0004 (7)	0.0364 (8)	−0.0034 (7)
O3C	0.0235 (7)	0.0074 (7)	0.0200 (7)	0.0002 (5)	0.0121 (5)	0.0015 (5)
O4C	0.0234 (7)	0.0110 (7)	0.0199 (6)	−0.0007 (5)	0.0132 (5)	0.0015 (5)
O5C	0.0161 (6)	0.0156 (7)	0.0152 (6)	0.0014 (5)	0.0089 (5)	−0.0011 (5)
O6C	0.0140 (6)	0.0144 (7)	0.0169 (6)	0.0020 (5)	0.0048 (5)	0.0038 (5)
O7C	0.0153 (6)	0.0124 (7)	0.0189 (7)	−0.0009 (5)	0.0020 (5)	−0.0031 (5)
N1C	0.0167 (7)	0.0108 (8)	0.0160 (7)	−0.0014 (6)	0.0093 (6)	0.0006 (6)
C1C	0.0251 (9)	0.0130 (10)	0.0233 (9)	0.0010 (8)	0.0153 (8)	−0.0015 (8)
C2C	0.0193 (8)	0.0096 (10)	0.0180 (9)	0.0022 (7)	0.0090 (7)	−0.0025 (7)
C3C	0.0154 (8)	0.0104 (9)	0.0127 (8)	−0.0013 (7)	0.0043 (7)	0.0002 (7)
C4C	0.0130 (8)	0.0106 (10)	0.0116 (8)	0.0009 (7)	0.0038 (6)	−0.0005 (7)
C5C	0.0133 (8)	0.0126 (10)	0.0128 (8)	−0.0002 (7)	0.0036 (6)	0.0004 (7)
C6C	0.0190 (9)	0.0104 (10)	0.0169 (8)	−0.0016 (7)	0.0090 (7)	−0.0007 (7)
C7C	0.0203 (9)	0.0167 (11)	0.0175 (9)	−0.0009 (7)	0.0117 (7)	−0.0020 (7)
C8C	0.0131 (8)	0.0130 (9)	0.0119 (8)	−0.0015 (7)	0.0034 (6)	0.0007 (7)
C9C	0.0138 (8)	0.0108 (9)	0.0131 (8)	0.0000 (7)	0.0053 (6)	−0.0002 (7)
C10C	0.0149 (8)	0.0111 (9)	0.0113 (8)	0.0005 (7)	0.0049 (6)	0.0009 (7)
C11C	0.0118 (8)	0.0128 (9)	0.0131 (8)	0.0012 (6)	0.0053 (6)	0.0000 (7)
C12C	0.0143 (8)	0.0118 (9)	0.0131 (8)	0.0006 (6)	0.0048 (6)	−0.0003 (7)
C13C	0.0141 (8)	0.0101 (9)	0.0142 (8)	−0.0008 (7)	0.0043 (6)	−0.0002 (7)
C14C	0.0137 (8)	0.0134 (9)	0.0136 (8)	0.0006 (7)	0.0062 (6)	0.0000 (7)
O1W	0.0246 (7)	0.0219 (8)	0.0212 (7)	−0.0058 (6)	0.0078 (6)	0.0013 (6)
O2W	0.0200 (7)	0.0395 (10)	0.0184 (7)	0.0018 (6)	0.0062 (5)	−0.0084 (6)

### Geometric parameters (Å, º)

**Table:**

O1—C1	1.450 (2)	O2B—C1B	1.438 (3)
O1—C2	1.378 (2)	O2B—C7B	1.369 (2)
O2—C1	1.428 (3)	O3B—H3B	1.01 (4)
O2—C7	1.360 (2)	O3B—C3B	1.343 (3)
O3—H3	0.96 (3)	O4B—C8B	1.258 (3)
O3—C3	1.347 (2)	O5B—H5B	0.94 (4)
O4—C8	1.255 (2)	O5B—C11B	1.424 (2)
O5—H5	0.84 (4)	O6B—H6B	0.89 (4)
O5—C11	1.421 (2)	O6B—C12B	1.437 (2)
O6—H6	0.85 (3)	O7B—H7B	0.86 (4)
O6—C12	1.433 (2)	O7B—C13B	1.431 (2)
O7—H7	0.89 (3)	N1B—H1BA	0.89 (4)
O7—C13	1.439 (2)	N1B—C8B	1.330 (3)
N1—H1	0.91 (4)	N1B—C9B	1.462 (2)
N1—C8	1.331 (3)	C1B—H1BB	0.9900
N1—C9	1.465 (2)	C1B—H1BC	0.9900
C1—H1A	0.9900	C2B—C3B	1.380 (3)
C1—H1B	0.9900	C2B—C7B	1.376 (3)
C2—C3	1.382 (3)	C3B—C4B	1.417 (3)
C2—C7	1.380 (3)	C4B—C5B	1.414 (3)
C3—C4	1.417 (3)	C4B—C8B	1.479 (2)
C4—C5	1.413 (3)	C5B—C6B	1.403 (3)
C4—C8	1.472 (2)	C5B—C10B	1.472 (3)
C5—C6	1.403 (3)	C6B—H6BA	0.9500
C5—C10	1.470 (3)	C6B—C7B	1.375 (3)
C6—H6A	0.9500	C9B—H9B	1.0000
C6—C7	1.375 (3)	C9B—C10B	1.515 (2)
C9—H9	1.0000	C9B—C11B	1.518 (3)
C9—C10	1.517 (2)	C10B—C14B	1.339 (3)
C9—C11	1.528 (2)	C11B—H11B	1.0000
C10—C14	1.336 (3)	C11B—C12B	1.527 (3)
C11—H11	1.0000	C12B—H12B	1.0000
C11—C12	1.520 (2)	C12B—C13B	1.527 (2)
C12—H12	1.0000	C13B—H13B	1.0000
C12—C13	1.533 (2)	C13B—C14B	1.507 (3)
C13—H13	1.0000	C14B—H14B	0.9500
C13—C14	1.500 (3)	O1C—C1C	1.439 (2)
C14—H14	0.9500	O1C—C2C	1.370 (2)
O1A—C1A	1.441 (2)	O2C—C1C	1.438 (3)
O1A—C2A	1.374 (2)	O2C—C7C	1.368 (2)
O2A—C1A	1.437 (3)	O3C—H3C	0.95 (5)
O2A—C7A	1.367 (2)	O3C—C3C	1.352 (2)
O3A—H3A	1.00 (4)	O4C—C8C	1.261 (2)
O3A—C3A	1.346 (2)	O5C—H5C	0.82 (3)
O4A—C8A	1.268 (2)	O5C—C11C	1.428 (2)
O5A—H5A	0.87 (3)	O6C—H6C	0.88 (4)
O5A—C11A	1.425 (2)	O6C—C12C	1.430 (2)
O6A—H6AA	0.82 (4)	O7C—H7C	0.86 (4)
O6A—C12A	1.442 (2)	O7C—C13C	1.443 (2)
O7A—H7A	0.83 (4)	N1C—H1C	0.91 (4)
O7A—C13A	1.439 (2)	N1C—C8C	1.323 (3)
N1A—H1AA	0.89 (3)	N1C—C9C	1.468 (2)
N1A—C8A	1.325 (3)	C1C—H1CA	0.9900
N1A—C9A	1.456 (2)	C1C—H1CB	0.9900
C1A—H1AB	0.9900	C2C—C3C	1.377 (3)
C1A—H1AC	0.9900	C2C—C7C	1.380 (3)
C2A—C3A	1.376 (3)	C3C—C4C	1.417 (3)
C2A—C7A	1.381 (3)	C4C—C5C	1.406 (3)
C3A—C4A	1.415 (3)	C4C—C8C	1.470 (2)
C4A—C5A	1.408 (3)	C5C—C6C	1.408 (3)
C4A—C8A	1.468 (2)	C5C—C10C	1.475 (3)
C5A—C6A	1.408 (3)	C6C—H6CA	0.9500
C5A—C10A	1.479 (3)	C6C—C7C	1.378 (3)
C6A—H6AB	0.9500	C9C—H9C	1.0000
C6A—C7A	1.379 (3)	C9C—C10C	1.515 (2)
C9A—H9A	1.0000	C9C—C11C	1.520 (3)
C9A—C10A	1.519 (2)	C10C—C14C	1.340 (3)
C9A—C11A	1.530 (3)	C11C—H11C	1.0000
C10A—C14A	1.335 (3)	C11C—C12C	1.523 (3)
C11A—H11A	1.0000	C12C—H12C	1.0000
C11A—C12A	1.516 (3)	C12C—C13C	1.536 (2)
C12A—H12A	1.0000	C13C—H13C	1.0000
C12A—C13A	1.536 (2)	C13C—C14C	1.501 (3)
C13A—H13A	1.0000	C14C—H14C	0.9500
C13A—C14A	1.503 (3)	O1W—H1WA	0.90 (4)
C14A—H14A	0.9500	O1W—H1WB	0.89 (5)
O1B—C1B	1.432 (2)	O2W—H2WA	0.90 (3)
O1B—C2B	1.374 (2)	O2W—H2WB	0.87 (4)
			
C2—O1—C1	105.17 (15)	C7B—O2B—C1B	105.90 (15)
C7—O2—C1	106.90 (16)	C3B—O3B—H3B	100 (2)
C3—O3—H3	101 (2)	C11B—O5B—H5B	104 (3)
C11—O5—H5	113 (3)	C12B—O6B—H6B	109 (2)
C12—O6—H6	108.5 (18)	C13B—O7B—H7B	108 (3)
C13—O7—H7	110 (2)	C8B—N1B—H1BA	113 (2)
C8—N1—H1	120 (2)	C8B—N1B—C9B	124.11 (16)
C8—N1—C9	123.65 (15)	C9B—N1B—H1BA	122 (2)
C9—N1—H1	115 (2)	O1B—C1B—O2B	108.01 (15)
O1—C1—H1A	110.2	O1B—C1B—H1BB	110.1
O1—C1—H1B	110.2	O1B—C1B—H1BC	110.1
O2—C1—O1	107.61 (16)	O2B—C1B—H1BB	110.1
O2—C1—H1A	110.2	O2B—C1B—H1BC	110.1
O2—C1—H1B	110.2	H1BB—C1B—H1BC	108.4
H1A—C1—H1B	108.5	O1B—C2B—C3B	127.77 (19)
O1—C2—C3	128.47 (19)	O1B—C2B—C7B	110.58 (17)
O1—C2—C7	110.32 (17)	C7B—C2B—C3B	121.64 (19)
C7—C2—C3	121.20 (19)	O3B—C3B—C2B	120.24 (19)
O3—C3—C2	120.81 (18)	O3B—C3B—C4B	123.17 (17)
O3—C3—C4	122.49 (17)	C2B—C3B—C4B	116.59 (19)
C2—C3—C4	116.70 (18)	C3B—C4B—C8B	118.08 (18)
C3—C4—C8	117.80 (17)	C5B—C4B—C3B	121.18 (16)
C5—C4—C3	121.24 (17)	C5B—C4B—C8B	120.70 (18)
C5—C4—C8	120.89 (17)	C4B—C5B—C10B	118.57 (16)
C4—C5—C10	118.47 (16)	C6B—C5B—C4B	120.42 (18)
C6—C5—C4	120.51 (18)	C6B—C5B—C10B	121.00 (18)
C6—C5—C10	120.97 (17)	C5B—C6B—H6BA	121.6
C5—C6—H6A	121.7	C7B—C6B—C5B	116.74 (18)
C7—C6—C5	116.53 (18)	C7B—C6B—H6BA	121.6
C7—C6—H6A	121.7	O2B—C7B—C2B	109.76 (18)
O2—C7—C2	109.92 (18)	O2B—C7B—C6B	126.81 (19)
O2—C7—C6	126.32 (18)	C6B—C7B—C2B	123.42 (17)
C6—C7—C2	123.75 (18)	O4B—C8B—N1B	121.09 (16)
O4—C8—N1	121.03 (17)	O4B—C8B—C4B	120.80 (18)
O4—C8—C4	119.94 (17)	N1B—C8B—C4B	118.10 (18)
N1—C8—C4	119.02 (17)	N1B—C9B—H9B	108.3
N1—C9—H9	108.2	N1B—C9B—C10B	111.40 (16)
N1—C9—C10	112.21 (15)	N1B—C9B—C11B	109.63 (15)
N1—C9—C11	109.71 (14)	C10B—C9B—H9B	108.3
C10—C9—H9	108.2	C10B—C9B—C11B	110.82 (16)
C10—C9—C11	110.07 (15)	C11B—C9B—H9B	108.3
C11—C9—H9	108.2	C5B—C10B—C9B	116.24 (17)
C5—C10—C9	116.86 (16)	C14B—C10B—C5B	123.32 (17)
C14—C10—C5	121.70 (16)	C14B—C10B—C9B	120.31 (17)
C14—C10—C9	121.44 (18)	O5B—C11B—C9B	107.93 (15)
O5—C11—C9	110.35 (14)	O5B—C11B—H11B	109.2
O5—C11—H11	108.1	O5B—C11B—C12B	111.42 (15)
O5—C11—C12	112.55 (15)	C9B—C11B—H11B	109.2
C9—C11—H11	108.1	C9B—C11B—C12B	109.82 (15)
C12—C11—C9	109.39 (14)	C12B—C11B—H11B	109.2
C12—C11—H11	108.1	O6B—C12B—C11B	111.14 (15)
O6—C12—C11	106.22 (14)	O6B—C12B—H12B	108.9
O6—C12—H12	109.8	O6B—C12B—C13B	107.57 (14)
O6—C12—C13	109.46 (14)	C11B—C12B—H12B	108.9
C11—C12—H12	109.8	C11B—C12B—C13B	111.24 (15)
C11—C12—C13	111.60 (15)	C13B—C12B—H12B	108.9
C13—C12—H12	109.8	O7B—C13B—C12B	107.13 (14)
O7—C13—C12	113.45 (14)	O7B—C13B—H13B	108.3
O7—C13—H13	107.2	O7B—C13B—C14B	111.25 (15)
O7—C13—C14	109.98 (15)	C12B—C13B—H13B	108.3
C12—C13—H13	107.2	C14B—C13B—C12B	113.29 (16)
C14—C13—C12	111.60 (15)	C14B—C13B—H13B	108.3
C14—C13—H13	107.2	C10B—C14B—C13B	124.99 (16)
C10—C14—C13	125.27 (16)	C10B—C14B—H14B	117.5
C10—C14—H14	117.4	C13B—C14B—H14B	117.5
C13—C14—H14	117.4	C2C—O1C—C1C	105.10 (16)
C2A—O1A—C1A	104.91 (15)	C7C—O2C—C1C	106.67 (16)
C7A—O2A—C1A	106.58 (15)	C3C—O3C—H3C	103 (3)
C3A—O3A—H3A	105 (2)	C11C—O5C—H5C	110 (2)
C11A—O5A—H5A	109 (2)	C12C—O6C—H6C	109 (2)
C12A—O6A—H6AA	108 (2)	C13C—O7C—H7C	106.1 (19)
C13A—O7A—H7A	107 (3)	C8C—N1C—H1C	117 (2)
C8A—N1A—H1AA	115 (2)	C8C—N1C—C9C	124.78 (15)
C8A—N1A—C9A	124.28 (15)	C9C—N1C—H1C	118 (2)
C9A—N1A—H1AA	118 (2)	O1C—C1C—H1CA	110.1
O1A—C1A—H1AB	110.1	O1C—C1C—H1CB	110.1
O1A—C1A—H1AC	110.1	O2C—C1C—O1C	107.84 (16)
O2A—C1A—O1A	107.88 (15)	O2C—C1C—H1CA	110.1
O2A—C1A—H1AB	110.1	O2C—C1C—H1CB	110.1
O2A—C1A—H1AC	110.1	H1CA—C1C—H1CB	108.5
H1AB—C1A—H1AC	108.4	O1C—C2C—C3C	127.27 (19)
O1A—C2A—C3A	127.45 (19)	O1C—C2C—C7C	111.40 (17)
O1A—C2A—C7A	111.22 (16)	C3C—C2C—C7C	121.32 (18)
C3A—C2A—C7A	121.30 (18)	O3C—C3C—C2C	120.07 (18)
O3A—C3A—C2A	120.67 (18)	O3C—C3C—C4C	123.41 (16)
O3A—C3A—C4A	122.38 (16)	C2C—C3C—C4C	116.50 (18)
C2A—C3A—C4A	116.96 (18)	C3C—C4C—C8C	117.46 (17)
C3A—C4A—C8A	117.54 (17)	C5C—C4C—C3C	121.64 (16)
C5A—C4A—C3A	121.26 (17)	C5C—C4C—C8C	120.86 (18)
C5A—C4A—C8A	121.18 (17)	C4C—C5C—C6C	120.46 (18)
C4A—C5A—C6A	120.50 (18)	C4C—C5C—C10C	117.64 (16)
C4A—C5A—C10A	118.17 (16)	C6C—C5C—C10C	121.82 (18)
C6A—C5A—C10A	121.32 (18)	C5C—C6C—H6CA	121.9
C5A—C6A—H6AB	121.8	C7C—C6C—C5C	116.15 (18)
C7A—C6A—C5A	116.35 (18)	C7C—C6C—H6CA	121.9
C7A—C6A—H6AB	121.8	O2C—C7C—C2C	108.97 (18)
O2A—C7A—C2A	109.16 (17)	O2C—C7C—C6C	127.17 (19)
O2A—C7A—C6A	127.30 (18)	C6C—C7C—C2C	123.85 (17)
C6A—C7A—C2A	123.52 (17)	O4C—C8C—N1C	121.55 (17)
O4A—C8A—N1A	120.63 (17)	O4C—C8C—C4C	119.37 (18)
O4A—C8A—C4A	119.93 (17)	N1C—C8C—C4C	119.08 (17)
N1A—C8A—C4A	119.44 (17)	N1C—C9C—H9C	108.6
N1A—C9A—H9A	108.1	N1C—C9C—C10C	111.19 (15)
N1A—C9A—C10A	112.74 (15)	N1C—C9C—C11C	108.90 (15)
N1A—C9A—C11A	110.01 (15)	C10C—C9C—H9C	108.6
C10A—C9A—H9A	108.1	C10C—C9C—C11C	110.94 (15)
C10A—C9A—C11A	109.54 (15)	C11C—C9C—H9C	108.6
C11A—C9A—H9A	108.1	C5C—C10C—C9C	116.99 (16)
C5A—C10A—C9A	118.22 (16)	C14C—C10C—C5C	122.51 (17)
C14A—C10A—C5A	122.58 (16)	C14C—C10C—C9C	120.49 (17)
C14A—C10A—C9A	119.20 (17)	O5C—C11C—C9C	107.69 (14)
O5A—C11A—C9A	111.93 (15)	O5C—C11C—H11C	108.9
O5A—C11A—H11A	108.0	O5C—C11C—C12C	111.65 (15)
O5A—C11A—C12A	111.71 (15)	C9C—C11C—H11C	108.9
C9A—C11A—H11A	108.0	C9C—C11C—C12C	110.64 (14)
C12A—C11A—C9A	109.10 (14)	C12C—C11C—H11C	108.9
C12A—C11A—H11A	108.0	O6C—C12C—C11C	112.11 (14)
O6A—C12A—C11A	106.78 (15)	O6C—C12C—H12C	109.1
O6A—C12A—H12A	109.3	O6C—C12C—C13C	107.37 (14)
O6A—C12A—C13A	110.60 (14)	C11C—C12C—H12C	109.1
C11A—C12A—H12A	109.3	C11C—C12C—C13C	110.04 (15)
C11A—C12A—C13A	111.51 (15)	C13C—C12C—H12C	109.1
C13A—C12A—H12A	109.3	O7C—C13C—C12C	109.32 (14)
O7A—C13A—C12A	107.89 (15)	O7C—C13C—H13C	108.6
O7A—C13A—H13A	108.6	O7C—C13C—C14C	108.14 (14)
O7A—C13A—C14A	108.86 (15)	C12C—C13C—H13C	108.6
C12A—C13A—H13A	108.6	C14C—C13C—C12C	113.34 (15)
C14A—C13A—C12A	114.07 (16)	C14C—C13C—H13C	108.6
C14A—C13A—H13A	108.6	C10C—C14C—C13C	125.07 (16)
C10A—C14A—C13A	125.19 (16)	C10C—C14C—H14C	117.5
C10A—C14A—H14A	117.4	C13C—C14C—H14C	117.5
C13A—C14A—H14A	117.4	H1WA—O1W—H1WB	109 (4)
C2B—O1B—C1B	105.30 (16)	H2WA—O2W—H2WB	107 (3)
			
O1—C2—C3—O3	−0.2 (3)	O1B—C2B—C3B—O3B	3.3 (3)
O1—C2—C3—C4	179.64 (18)	O1B—C2B—C3B—C4B	−177.19 (17)
O1—C2—C7—O2	0.2 (2)	O1B—C2B—C7B—O2B	−0.7 (2)
O1—C2—C7—C6	179.57 (19)	O1B—C2B—C7B—C6B	177.95 (18)
O3—C3—C4—C5	−179.83 (17)	O3B—C3B—C4B—C5B	178.08 (17)
O3—C3—C4—C8	3.2 (3)	O3B—C3B—C4B—C8B	0.6 (3)
O5—C11—C12—O6	−66.93 (18)	O5B—C11B—C12B—O6B	−60.52 (19)
O5—C11—C12—C13	173.83 (14)	O5B—C11B—C12B—C13B	179.64 (14)
O6—C12—C13—O7	156.75 (15)	O6B—C12B—C13B—O7B	148.66 (15)
O6—C12—C13—C14	−78.34 (18)	O6B—C12B—C13B—C14B	−88.28 (18)
O7—C13—C14—C10	118.47 (19)	O7B—C13B—C14B—C10B	118.34 (19)
N1—C9—C10—C5	34.0 (2)	N1B—C9B—C10B—C5B	37.0 (2)
N1—C9—C10—C14	−146.53 (17)	N1B—C9B—C10B—C14B	−147.00 (17)
N1—C9—C11—O5	−58.02 (19)	N1B—C9B—C11B—O5B	−59.61 (19)
N1—C9—C11—C12	177.64 (14)	N1B—C9B—C11B—C12B	178.75 (15)
C1—O1—C2—C3	−179.75 (19)	C1B—O1B—C2B—C3B	−176.65 (19)
C1—O1—C2—C7	1.5 (2)	C1B—O1B—C2B—C7B	4.6 (2)
C1—O2—C7—C2	−1.9 (2)	C1B—O2B—C7B—C2B	−3.5 (2)
C1—O2—C7—C6	178.8 (2)	C1B—O2B—C7B—C6B	177.91 (19)
C2—O1—C1—O2	−2.6 (2)	C2B—O1B—C1B—O2B	−6.6 (2)
C2—C3—C4—C5	0.3 (3)	C2B—C3B—C4B—C5B	−1.4 (3)
C2—C3—C4—C8	−176.66 (16)	C2B—C3B—C4B—C8B	−178.91 (16)
C3—C2—C7—O2	−178.66 (17)	C3B—C2B—C7B—O2B	−179.54 (17)
C3—C2—C7—C6	0.7 (3)	C3B—C2B—C7B—C6B	−0.9 (3)
C3—C4—C5—C6	2.1 (3)	C3B—C4B—C5B—C6B	0.8 (3)
C3—C4—C5—C10	−175.14 (16)	C3B—C4B—C5B—C10B	−179.02 (16)
C3—C4—C8—O4	1.6 (3)	C3B—C4B—C8B—O4B	5.5 (3)
C3—C4—C8—N1	−179.57 (16)	C3B—C4B—C8B—N1B	−175.36 (16)
C4—C5—C6—C7	−3.0 (3)	C4B—C5B—C6B—C7B	−0.2 (3)
C4—C5—C10—C9	−21.2 (2)	C4B—C5B—C10B—C9B	−21.3 (2)
C4—C5—C10—C14	159.35 (18)	C4B—C5B—C10B—C14B	162.86 (18)
C5—C4—C8—O4	−175.36 (16)	C5B—C4B—C8B—O4B	−172.00 (16)
C5—C4—C8—N1	3.4 (3)	C5B—C4B—C8B—N1B	7.1 (3)
C5—C6—C7—O2	−179.04 (19)	C5B—C6B—C7B—O2B	178.59 (18)
C5—C6—C7—C2	1.7 (3)	C5B—C6B—C7B—C2B	0.2 (3)
C5—C10—C14—C13	−179.43 (16)	C5B—C10B—C14B—C13B	173.39 (16)
C6—C5—C10—C9	161.62 (17)	C6B—C5B—C10B—C9B	158.87 (17)
C6—C5—C10—C14	−17.9 (3)	C6B—C5B—C10B—C14B	−17.0 (3)
C7—O2—C1—O1	2.7 (2)	C7B—O2B—C1B—O1B	6.3 (2)
C7—C2—C3—O3	178.45 (17)	C7B—C2B—C3B—O3B	−178.04 (17)
C7—C2—C3—C4	−1.7 (3)	C7B—C2B—C3B—C4B	1.5 (3)
C8—N1—C9—C10	−30.9 (2)	C8B—N1B—C9B—C10B	−34.1 (3)
C8—N1—C9—C11	−153.55 (16)	C8B—N1B—C9B—C11B	−157.12 (17)
C8—C4—C5—C6	178.98 (16)	C8B—C4B—C5B—C6B	178.22 (16)
C8—C4—C5—C10	1.8 (3)	C8B—C4B—C5B—C10B	−1.6 (3)
C9—N1—C8—O4	−168.63 (16)	C9B—N1B—C8B—O4B	−168.45 (17)
C9—N1—C8—C4	12.6 (3)	C9B—N1B—C8B—C4B	12.4 (3)
C9—C10—C14—C13	1.1 (3)	C9B—C10B—C14B—C13B	−2.3 (3)
C9—C11—C12—O6	56.11 (18)	C9B—C11B—C12B—O6B	59.00 (19)
C9—C11—C12—C13	−63.13 (18)	C9B—C11B—C12B—C13B	−60.84 (19)
C10—C5—C6—C7	174.14 (16)	C10B—C5B—C6B—C7B	179.65 (16)
C10—C9—C11—O5	178.03 (14)	C10B—C9B—C11B—O5B	177.02 (14)
C10—C9—C11—C12	53.69 (19)	C10B—C9B—C11B—C12B	55.38 (19)
C11—C9—C10—C5	156.46 (15)	C11B—C9B—C10B—C5B	159.39 (15)
C11—C9—C10—C14	−24.0 (2)	C11B—C9B—C10B—C14B	−24.7 (2)
C11—C12—C13—O7	−85.96 (19)	C11B—C12B—C13B—O7B	−89.41 (18)
C11—C12—C13—C14	38.95 (19)	C11B—C12B—C13B—C14B	33.7 (2)
C12—C13—C14—C10	−8.4 (2)	C12B—C13B—C14B—C10B	−2.4 (2)
O1A—C2A—C3A—O3A	−0.5 (3)	O1C—C2C—C3C—O3C	0.4 (3)
O1A—C2A—C3A—C4A	179.59 (17)	O1C—C2C—C3C—C4C	178.95 (18)
O1A—C2A—C7A—O2A	0.6 (2)	O1C—C2C—C7C—O2C	1.2 (2)
O1A—C2A—C7A—C6A	179.06 (17)	O1C—C2C—C7C—C6C	−177.97 (19)
O3A—C3A—C4A—C5A	−178.42 (16)	O3C—C3C—C4C—C5C	176.47 (16)
O3A—C3A—C4A—C8A	2.7 (3)	O3C—C3C—C4C—C8C	−1.3 (3)
O5A—C11A—C12A—O6A	−62.49 (18)	O5C—C11C—C12C—O6C	−62.17 (19)
O5A—C11A—C12A—C13A	176.59 (14)	O5C—C11C—C12C—C13C	178.41 (14)
O6A—C12A—C13A—O7A	147.36 (15)	O6C—C12C—C13C—O7C	153.23 (15)
O6A—C12A—C13A—C14A	−91.56 (19)	O6C—C12C—C13C—C14C	−86.06 (18)
O7A—C13A—C14A—C10A	124.37 (19)	O7C—C13C—C14C—C10C	116.59 (19)
N1A—C9A—C10A—C5A	26.8 (2)	N1C—C9C—C10C—C5C	35.9 (2)
N1A—C9A—C10A—C14A	−153.50 (17)	N1C—C9C—C10C—C14C	−143.25 (17)
N1A—C9A—C11A—O5A	−50.89 (19)	N1C—C9C—C11C—O5C	−61.17 (18)
N1A—C9A—C11A—C12A	−175.04 (14)	N1C—C9C—C11C—C12C	176.56 (14)
C1A—O1A—C2A—C3A	−179.61 (19)	C1C—O1C—C2C—C3C	179.72 (19)
C1A—O1A—C2A—C7A	2.5 (2)	C1C—O1C—C2C—C7C	−1.0 (2)
C1A—O2A—C7A—C2A	−3.5 (2)	C1C—O2C—C7C—C2C	−0.8 (2)
C1A—O2A—C7A—C6A	178.11 (18)	C1C—O2C—C7C—C6C	178.3 (2)
C2A—O1A—C1A—O2A	−4.62 (19)	C2C—O1C—C1C—O2C	0.5 (2)
C2A—C3A—C4A—C5A	1.5 (3)	C2C—C3C—C4C—C5C	−2.0 (3)
C2A—C3A—C4A—C8A	−177.38 (16)	C2C—C3C—C4C—C8C	−179.76 (16)
C3A—C2A—C7A—O2A	−177.39 (17)	C3C—C2C—C7C—O2C	−179.49 (18)
C3A—C2A—C7A—C6A	1.1 (3)	C3C—C2C—C7C—C6C	1.3 (3)
C3A—C4A—C5A—C6A	1.6 (3)	C3C—C4C—C5C—C6C	3.2 (3)
C3A—C4A—C5A—C10A	−178.01 (16)	C3C—C4C—C5C—C10C	−173.58 (16)
C3A—C4A—C8A—O4A	0.2 (3)	C3C—C4C—C8C—O4C	4.6 (3)
C3A—C4A—C8A—N1A	179.32 (16)	C3C—C4C—C8C—N1C	−175.10 (15)
C4A—C5A—C6A—C7A	−3.2 (3)	C4C—C5C—C6C—C7C	−2.1 (3)
C4A—C5A—C10A—C9A	−15.2 (2)	C4C—C5C—C10C—C9C	−26.4 (2)
C4A—C5A—C10A—C14A	165.18 (17)	C4C—C5C—C10C—C14C	152.74 (18)
C5A—C4A—C8A—O4A	−178.64 (16)	C5C—C4C—C8C—O4C	−173.22 (16)
C5A—C4A—C8A—N1A	0.5 (3)	C5C—C4C—C8C—N1C	7.1 (3)
C5A—C6A—C7A—O2A	−179.85 (18)	C5C—C6C—C7C—O2C	−179.17 (19)
C5A—C6A—C7A—C2A	2.0 (3)	C5C—C6C—C7C—C2C	−0.1 (3)
C5A—C10A—C14A—C13A	178.01 (16)	C5C—C10C—C14C—C13C	178.13 (17)
C6A—C5A—C10A—C9A	165.26 (16)	C6C—C5C—C10C—C9C	156.83 (17)
C6A—C5A—C10A—C14A	−14.4 (3)	C6C—C5C—C10C—C14C	−24.0 (3)
C7A—O2A—C1A—O1A	5.1 (2)	C7C—O2C—C1C—O1C	0.2 (2)
C7A—C2A—C3A—O3A	177.13 (16)	C7C—C2C—C3C—O3C	−178.76 (16)
C7A—C2A—C3A—C4A	−2.8 (3)	C7C—C2C—C3C—C4C	−0.2 (3)
C8A—N1A—C9A—C10A	−27.3 (2)	C8C—N1C—C9C—C10C	−26.4 (2)
C8A—N1A—C9A—C11A	−149.93 (17)	C8C—N1C—C9C—C11C	−148.98 (17)
C8A—C4A—C5A—C6A	−179.63 (16)	C8C—C4C—C5C—C6C	−179.08 (16)
C8A—C4A—C5A—C10A	0.8 (3)	C8C—C4C—C5C—C10C	4.1 (2)
C9A—N1A—C8A—O4A	−166.72 (17)	C9C—N1C—C8C—O4C	−174.15 (17)
C9A—N1A—C8A—C4A	14.2 (3)	C9C—N1C—C8C—C4C	5.5 (3)
C9A—C10A—C14A—C13A	−1.6 (3)	C9C—C10C—C14C—C13C	−2.8 (3)
C9A—C11A—C12A—O6A	61.79 (17)	C9C—C11C—C12C—O6C	57.75 (19)
C9A—C11A—C12A—C13A	−59.13 (19)	C9C—C11C—C12C—C13C	−61.66 (18)
C10A—C5A—C6A—C7A	176.34 (16)	C10C—C5C—C6C—C7C	174.59 (16)
C10A—C9A—C11A—O5A	−175.35 (15)	C10C—C9C—C11C—O5C	176.14 (14)
C10A—C9A—C11A—C12A	60.50 (19)	C10C—C9C—C11C—C12C	53.87 (19)
C11A—C9A—C10A—C5A	149.70 (16)	C11C—C9C—C10C—C5C	157.25 (15)
C11A—C9A—C10A—C14A	−30.6 (2)	C11C—C9C—C10C—C14C	−21.9 (2)
C11A—C12A—C13A—O7A	−93.98 (18)	C11C—C12C—C13C—O7C	−84.50 (18)
C11A—C12A—C13A—C14A	27.1 (2)	C11C—C12C—C13C—C14C	36.21 (19)
C12A—C13A—C14A—C10A	3.8 (3)	C12C—C13C—C14C—C10C	−4.8 (3)

### Hydrogen-bond geometry (Å, º)

**Table:**

*D*—H···*A*	*D*—H	H···*A*	*D*···*A*	*D*—H···*A*
O3—H3···O4	0.96 (3)	1.57 (4)	2.4842 (19)	158 (3)
O5—H5···O7*A*^i^	0.84 (4)	1.88 (4)	2.698 (2)	166 (4)
O6—H6···O3*A*^ii^	0.85 (3)	1.98 (3)	2.745 (2)	150 (3)
O7—H7···O7*C*^iii^	0.89 (3)	1.89 (3)	2.768 (2)	170 (3)
N1—H1···O4*B*^i^	0.91 (4)	2.25 (4)	3.105 (2)	157 (3)
O3*A*—H3*A*···O4*A*	1.00 (4)	1.55 (4)	2.4656 (19)	151 (4)
O5*A*—H5*A*···O7	0.87 (3)	2.23 (3)	3.050 (2)	157 (3)
O6*A*—H6*AA*···O1^ii^	0.82 (4)	2.15 (4)	2.906 (2)	153 (3)
O7*A*—H7*A*···O1*A*^iv^	0.83 (4)	2.29 (4)	2.901 (2)	131 (3)
N1*A*—H1*AA*···O4*C*	0.89 (3)	1.91 (3)	2.796 (2)	171 (3)
O3*B*—H3*B*···O4*B*	1.01 (4)	1.57 (4)	2.537 (2)	159 (4)
O5*B*—H5*B*···O2*W*^v^	0.94 (4)	1.80 (4)	2.701 (2)	159 (4)
O6*B*—H6*B*···O6^vi^	0.89 (4)	2.03 (4)	2.8857 (19)	163 (3)
O7*B*—H7*B*···O5^vii^	0.86 (4)	1.86 (4)	2.714 (2)	175 (4)
N1*B*—H1*BA*···O4^v^	0.89 (4)	1.99 (4)	2.870 (2)	171 (3)
O3*C*—H3*C*···O4*C*	0.95 (5)	1.59 (5)	2.4797 (18)	154 (5)
O5*C*—H5*C*···O6*B*	0.82 (3)	2.01 (4)	2.829 (2)	175 (3)
O6*C*—H6*C*···O6*A*^vi^	0.88 (4)	1.93 (3)	2.7893 (19)	165 (3)
O7*C*—H7*C*···O3*C*^vii^	0.86 (4)	1.93 (4)	2.767 (2)	162 (3)
N1*C*—H1*C*···O4*A*	0.91 (4)	2.03 (4)	2.911 (2)	164 (3)
O1*W*—H1*WA*···O7*B*^i^	0.90 (4)	1.94 (4)	2.835 (2)	173 (4)
O1*W*—H1*WB*···O5*A*^vii^	0.89 (5)	2.22 (5)	3.036 (2)	153 (4)
O2*W*—H2*WA*···O1*W*	0.90 (3)	1.94 (3)	2.833 (2)	167 (3)
O2*W*—H2WB···O6*C*	0.87 (4)	2.00 (4)	2.869 (2)	172 (3)

Symmetry codes: (i) *x*, *y*, *z*−1; (ii) −*x*, *y*−1/2, −*z*+1; (iii) −*x*+1, *y*−1/2, −*z*+1; (iv) −*x*, *y*−1/2, −*z*+2; (v) *x*, *y*, *z*+1; (vi) −*x*, *y*+1/2, −*z*+1; (vii) −*x*+1, *y*+1/2, −*z*+1.

## References

[bb1] Agilent (2012). *CrysAlis PRO* Agilent Technologies, Yarnton, England.

[bb2] Bi, Y.-R., Yung, K.-H. & Wong, Y.-S. (1998). *Plant. Sci.* **135**, 103–108.

[bb3] Ceriotti, G. (1967*a*). *Nature*, **213**, 595–596. 10.1038/213595a0 5340258

[bb4] Ceriotti, G. (1967*b*). *Tumori*, **53**, 437–445. 10.1177/030089166705300501 6076975

[bb5] Ceriotti, G., Spandrio, L. & Gazzaniga, A. (1967). *Tumori*, **53**, 359–371. 10.1177/030089166705300406 6069778

[bb6] Flack, H. D. (1983). *Acta Cryst.* A**39**, 876–881.

[bb7] Immirzi, A. & Fuganti, C. (1972). *J. Chem. Soc. Chem. Commun.* p. 240a.

[bb8] Kornienko, A. & Evidente, A. (2008). *Chem. Rev.* **108**, 1982–2014. 10.1021/cr078198u PMC2856661 18489166

[bb9] McNulty, J., Thorat, A., Vurgun, N., Nair, J. J., Makaji, E., Crankshaw, D. J., Holloway, A. C. & Pandey, S. (2011). *J. Nat. Prod.* **74**, 106–108. 10.1021/np100657w 21105682

[bb10] Savona, G., Piozzi, F., Marino, M. L., Knight, J. & Mays, M. J. (1970). *J. Chem. Soc. Chem. Commun.* p. 1006a.

[bb11] Sheldrick, G. M. (2008). *Acta Cryst.* A**64**, 112–122. 10.1107/S0108767307043930 18156677

